# *Gm*CBP60b Plays Both Positive and Negative Roles in Plant Immunity

**DOI:** 10.3390/ijms25010378

**Published:** 2023-12-27

**Authors:** Mei-Yan Ye, Hu-Jiao Lan, Jian-Zhong Liu

**Affiliations:** 1 College of Life Sciences, Zhejiang Normal University, Jinhua 321004, China; a19557861320@163.com (M.-Y.Y.); 18659351751@163.com (H.-J.L.); 2 Zhejiang Provincial Key Laboratory of Biotechnology on Specialty Economic Plants, Zhejiang Normal University, Jinhua 321004, China

**Keywords:** *Gm*CBP60b.1/2, VIGS, soybean, immunity

## Abstract

CBP60b (CALMODULIN-BINDING PROTEIN 60b) is a member of the CBP60 transcription factor family. In Arabidopsis, *At*CBP60b not only regulates growth and development but also activates the transcriptions in immune responses. So far, CBP60b has only been studied extensively in the model plant Arabidopsis and rarely in crops. In this study, *Bean pod mottle virus* (BPMV)-mediated gene silencing (BPMV-VIGS) was used to silence *GmCBP60b.1/2* in soybean plants. The silencing of *GmCBP60b.1/2* resulted in typical autoimmunity, such as dwarfism and enhanced resistance to both *Soybean mosaic virus* (SMV) and *Pseudomonas syringae* pv. glycinea (*Psg*). To further understand the roles of *Gm*CBP60b in immunity and circumvent the recalcitrance of soybean transformation, we generated transgenic tobacco lines that overexpress *GmCBP60b.1*. The overexpression of *GmCBP60b.1* also resulted in autoimmunity, including spontaneous cell death on the leaves, highly induced expression of PATHOGENESIS-RELATED (*PR*) genes, significantly elevated accumulation of defense hormone salicylic acid (SA), and significantly enhanced resistance to *Pst* DC3000 (*Pseudomonas syrangae* pv. tomato DC3000). The transient coexpression of a luciferase reporter gene driven by the promoter of soybean SYSTEMIC ACQUIRED RESISTANCE DEFICIENT 1 (*GmSARD1*) (*ProGmSARD1::LUC*), together with *GmCBP60b.1* driven by the 35S promoter, led to the activation of the LUC reporter gene, suggesting that *Gm*CBP60b.1 could bind to the core (A/T)AATT motifs within the promoter region of *GmSARD1* and, thus, activate the expression of the LUC reporter. Taken together, our results indicate that *Gm*CBP60b.1/2 play both positive and negative regulatory roles in immune responses. These results also suggest that the function of CBP60b is conserved across plant species.

## 1. Introduction

CALMODULIN-BINDING PROTEIN 60 (CBP60)-like proteins comprise a family of eight members of atypical transcription factors in Arabidopsis: CBP60a–CBP60g and SAR Deficient 1 (SARD1) [1]. The functional roles of CBP60g and SARD1 in immunity have been extensively studied [2,3,4,5,6,7]. They regulate the expression of defense-related genes by directly binding to their promoters [4]. These genes include those involved in SA and N-HYDROXY-PIPECOLIC ACID (NHP) biosynthesis, as well as in PAMP-triggered immunity (PTI), effector-triggered immunity (ETI), and systemic acquired resistance (SAR) pathways [4,7,8,9]. Unlike other CBP60s, SARD1 does not need to bind CALMODULIN (CaM) for its function [4]. SARD1 activates ISOCHORISMATE SYNTHASE 1 (*ICS1*) expression by binding a GAAATTT motif in its promoter [8]. The simultaneous loss of functions of SARD1 and CBP60g almost completely blocked pathogen-induced SA accumulation, leading to a reduced resistance to various pathogens [2,4]. Contrary to CBP60g or SARD1, CBP60a has been shown to play a negative role in immunity [10]. Recently, two groups provided solid evidence that CBP60b plays both positive and negative roles in Arabidopsis immunity [11,12]. *CBP60b* is a constitutively expressed gene [13], whereas *SARD1* and *CBP60g* are pathogen-induced genes [2,4]. The loss of function of CBP60b constitutively activated ENHANCED DISEASE SUSCEPTIBILITY 1 (EDS1)- and PHYTOALEXIN DEFICIENT 4 (PAD4)-dependent immunities [11,12], whereas the overexpression of CBP60b activated EDS1- and PAD4-independent immunities [11]. The authors proposed that CBP60b might be indispensable for the expression of a negative regulator of Toll-interleukin-1 receptor–nucleotide-binding leucine-rich repeat (TNL)-mediated immune signaling or that CBP60b might be monitored and guarded by a TNL directly or indirectly, and the loss of its function activates this unidentified TNL and, thus, results in ETI [11,12]. This postulation is supported by the partial requirement for TNL SNC1 (SUPPRESSOR OF NPR1-1, CONSTITUTIVE 1) in the autoimmunity of a *cbp60b* mutant [11]. The enhanced autoimmunity observed in the *cbp60bcbp60g-1* double mutant compared to either single mutant suggests that CBP60b and CBP60g play partially redundant roles in the transcriptional activation of the putative guardee/decoy guarded by unknown TNL [12].

Although extensively studied in model plant Arabidopsis, the functions of CBP60 family members have not been investigated in other plant species, especially in crops. In this report, both *GmCBP60b1* and *GmCBP60b2* (referred to as *GmCBP60b1/2* hereafter) were simultaneously silenced in soybeans via virus-induced gene silencing (VIGS) mediated by BPMV (*Bean pod mottle virus*), referred to as BPMV-VIGS hereafter. To circumvent the recalcitrance of soybeans in transformation, transgenic *Nicotiana tabacum* plants overexpressing *GmCBP60b1* were generated. Consistent with the observations in Arabidopsis, either silencing *GmCBP60b1/2* in soybeans or overexpressing *GmCBP60b1* in *N. tabacum* activated immune responses, indicating that *GmCBP60b1/2* play both positive and negative roles in immunity. A transient luciferase reporter assay demonstrated that *Gm*CBP60b.1 could bind to the core motif (A/T)AATT within the promoter region of *GmSARD1* and, thus, activate the expression of the LUC reporter, suggesting that the *Gm*CBP60b1 protein binds to a similar core motif as in Arabidopsis. Consistent with its role as a transcription factor, *Gm*CBP60b1 was exclusively localized in nuclei. Taken together, our results indicate that the function of CBP60b is conserved in regulating immunity across plant species.

## 2. Results

### 2.1. Silencing GmCBP60b.1 and GmCBP60b.2 Simultaneously Results in Stunted Stature and Enhanced Disease Resistance in Soybean Plants

It has been reported that CBP601b plays a critical role in immunity in the model plant Arabidopsis [11,12]. However, the function of CBP601b homologues in soybean immunity has not been previously investigated. To this end, the BPMV-VIGS system was used to investigate the functions of *Gm*CBP60bs. Soybeans are a paleopolyploid, in which 75% of the genes in their genome have two copies [14]. Consistent with this, two homologous *GmCBP60b*s sharing 97% identity at the nucleotide level were identified in the soybean genome, and they were referred to as *GmCBP60b.1* and *GmCBP60b.2*. *GmCBP60b.1* and *GmCBP60b.2* share 67% and 66% similarity with Arabidopsis *CBP60b*, respectively. To overcome the functional redundancy of these two genes, a 303 bp fragment amplified from *GmCBP60b.1* sharing 98% identity at the nucleotide level with the corresponding region of *CBP60b.2* was cloned into the BPMV-2 vector [15,16]. To infect the soybean plants, the plasmid DNA of the BPMV1 vector was co-bombarded with plasmid DNA of either the BPMV2 empty vector (referred to as BPMV-0) or BPMV-*GmCBP60b.1/2* (referred to as BPMV-*GmCBP60b.1/2*) into the first two true leaves of 7-day-old soybean seedlings. The viral symptoms were visible on the upper systemic leaves of the bombarded plants at 15 to 20 days post-bombardment (dpb). The systemic leaves with viral symptoms were ground in a phosphate buffer (pH 7.0). The liquid phase (sap), containing numerous viral particles, was collected and stored at −80 °C for subsequent batch inoculations.

**Figure 1:**
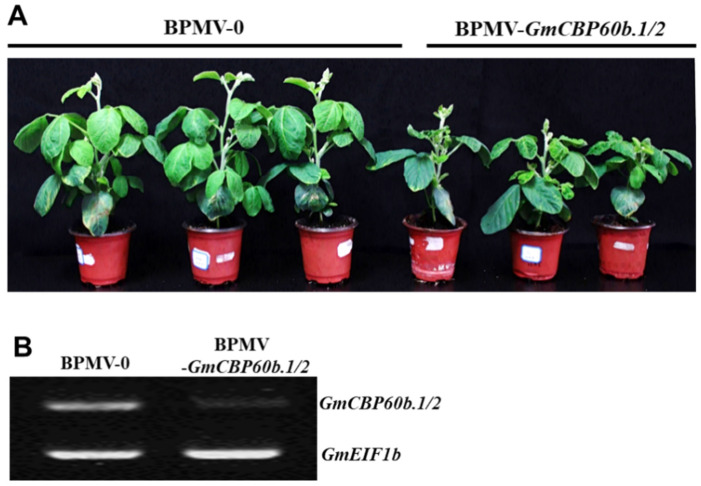
Silencing *GmCBP60b.1/2* simultaneously in soybean results in dwarfed phenotype. (**A**) The dwarfed phenotype of the *GmCBP60b.1/2*-silenced soybean plant; (**B**) RT-PCR analysis showed that *GmCBP60b.1/2* were indeed silenced. *GmELF1b* was used as an internal reference gene.

The 7-day-old seedlings were rub-inoculated with either the BPMV-0 or BPMV-*GmCBP60b.1/2* sap. At 20 days post-inoculation (dpi), the plants inoculated with BPMV-*GmCBP60b.1/2* sap displayed a stunted phenotype compared with the those inoculated with BPMV-0 sap (Figure 1A), indicating that simultaneously silencing both *GmCBP60b.1* and *GmCBP60b.2* dramatically reduced the sizes of the soybean plants. This stunted phenotype is similar to those plants with autoimmunity. Because the *cbp60b* mutant in Arabidopsis exhibited autoimmunity, it suggests that defense responses are similarly activated in the *GmCBP60b.1/2*-silenced plants. An RT-PCR analysis indicated that the transcript level of *GmCBP60b.1/2* was indeed significantly reduced in the *GmCBP60b.1/2*-silenced plants compared with the BPMV-0 plants (Figure 1B).

### 2.2. Silencing GmCBP60b.1/2 Enhances the Resistance of Soybean Plants to Both SMV-N-GUS and Pseudomonas syringae pv. glycinea (Psg)

**Figure 2:**
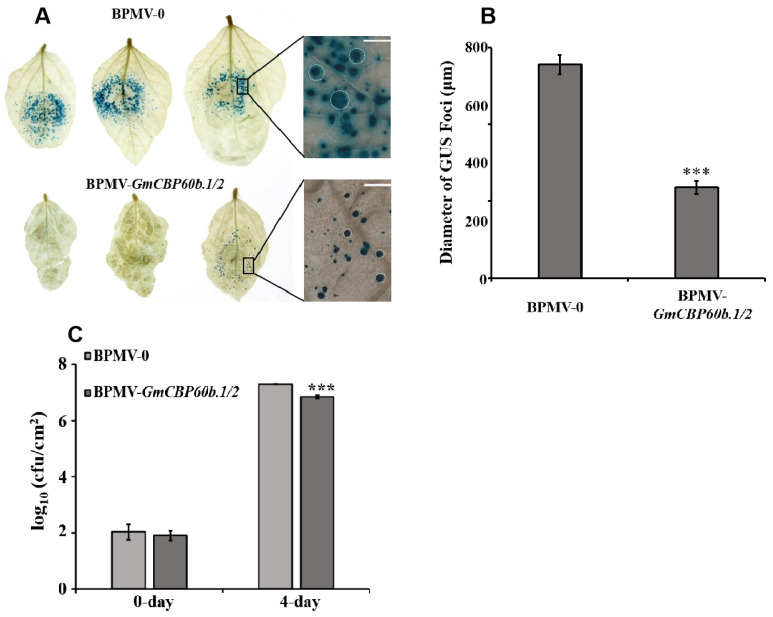
Silencing *GmCBP60b.1/2* results in enhanced resistance to SMV-N-GUS and *Psg*. (**A**) Comparison of the blue foci of SMV-N-GUS on the leaves of the BPMV-0 plants with those on the *GmCBP60b.1/2*-silenced plants at 3 days post-infection (dpi). The enlarged images were taken under a dissecting microscope (right). Scare bars = 1 mm. (**B**) Comparison of the diameters of SMV-N-GUS foci on the leaves of the BPMV-0 plants with those on the *GmCBP60b.1/2*-silenced plants under a dissecting microscope. At least 30 GUS foci were randomly picked for diameter measurement for each infected leaf, and 4 independent infected leaves were measured. Student’s *t*-test (***, *p* < 0.001). (**C**) Comparison of colony-forming units (cfu) between the BPMV-0 plants and the *GmCBP60b.1/2*-silenced plants at 0 dpi and 4 dpi post-*Psg* infection. The detailed procedure is described in Section 4. Student’s *t*-test (***, *p* < 0.001).

To test whether the stunted phenotype is associated with activated defense responses, we firstly inoculated BPMV-0 and BPMV-*GmCBP60b1/2* plants with the SMV N strain fused with the GUS reporter gene (SMV-N-GUS) [17] via biolistic bombardment. The successful infection of SMV-N-GUS can be visualized by the presence of blue GUS foci. The intensities and diameters of the GUS foci reflect replication efficiency and cell-to-cell movement, respectively. As shown in Figure 2A, much fewer blue GUS foci were observed on the infected leaves of the *GmCBP60b1/2*-silenced plants than on the infected vector control leaves. In addition, the diameter of the GUS foci was significantly smaller on the infected leaves of the *GmCBP60b.1/2*-silenced plants than on the infected vector control leaves (Figure 2B), indicating that silencing *GmCBP60b.1/2* enhances resistance to SMV-N-GUS.

Next, we examined the effect of *GmCBP60b.1/2* silencing on resistance against the bacterial pathogen *Pseudomonas syrangae* pv. glycinea (*Psg*). As shown in Figure 2C, the multiplication of *Psg* on the leaves of the *GmCBP60b.1/2*-silenced plants was drastically reduced compared with that on the BPMV-0 leaves. These results demonstrate that *GmCBP60b.1/2* play a negative role in disease resistance.

### 2.3. Overexpression of GmCBP60b.1 in Nicotiana tabacum Activates Immune Responses and Enhances Disease Resistance

**Figure 3:**
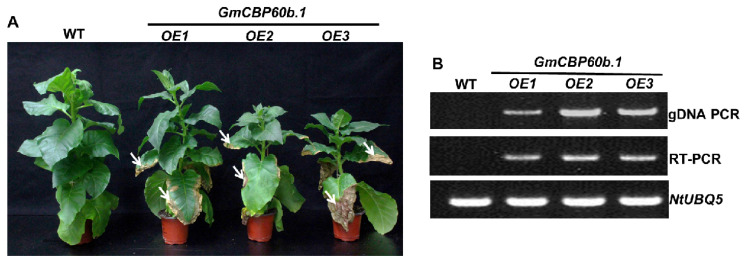
Overexpression of *GmCBP60b.1* leads to dwarfism and spontaneous cell death. (**A**) Comparison of phenotype between wild-type tobacco and *GmCBP60b.1*-overexpressing tobacco plants (65 days old). White arrows point to the regions with cell death. (**B**) The authenticity of the *GmCBP60b.1*-overexpressing lines was verified by both genomic PCR and RT-PCR. *NtUBQ5* was used as the internal reference gene.

To further understand the function of *Gm*CBP60b in immunity and overcome the difficulty of soybean transformation, we generated transgenic tobacco lines that overexpress *GmCBP60b.1*. The tobacco *GmCBP60b.1*-overexpressing lines displayed a stunted stature compared to the wide-type (WT) plants (Figure 3A). To our surprise, spontaneous cell death was observed on the leaves of the *GmCBP60b.1*-overexpressing tobacco lines (Figure 3A, see arrows), suggesting that the immune responses might be activated. The authenticity of the transgenic plants was verified by both genomic PCR and an RT-PCR analysis (Figure 3B).

**Figure 4:**
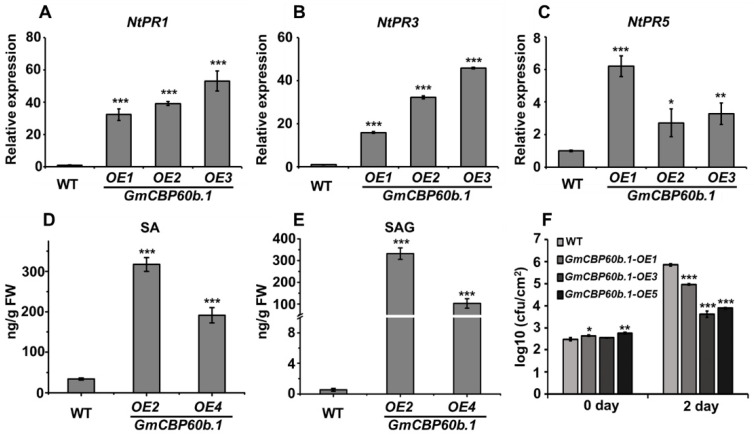
Overexpression of *GmCBP60b.1* leads to elevated SA accumulation levels, induced *PR* gene expression, and enhance resistance against *Pst* DC3000 in tobacco. (**A**–**C**) The expression of *PR1*, *PR3*, and *PR5* was significantly induced in the *GmCBP60b.1*-overexpressing plants, determined using RT-qPCR analysis. Student’s *t*-test (*, *p* < 0.05; **, *p* < 0.05; ***, *p* < 0.001). Comparison of free SA content (**D**) and SAG content (**E**) in leaves of wild-type tobacco plants and the *GmCBP60b.1*-overexpressing plants. (**F**) Comparison of colony-forming units between WT plants and the *GmCBP60b.1*-overexppressing plants at 0 dpi and 2 dpi of *Pst* DC3000 infection. Student’s *t*-test (***, *p* < 0.001).

To confirm that the stunted phenotype and cell death are indeed caused by the activated immunity, we firstly analyzed the expression of multiple *PR* genes. As shown in Figure 4A–C, the expressions of *NtPR1*, *NtPR3*, and *NtPR5* were hugely induced in the leaves of the *GmCBP60b.1*-overexpressing plants compared with the WT leaves. In addition, the accumulation levels of both free SA and conjugated SA were significantly elevated in the *GmCBP60b.1*-overexpressing plants relative to the WT plants (Figure 4D,E). Taken together, these results demonstrate that the overexpression of *GmCBP60b.1* activated the immune responses in tobacco.

Activated immune responses are usually correlated with enhanced disease resistance. To examine whether the activated immunity observed in the *GmCBP60b.1*-overexpressing plants results in enhanced resistance, we inoculated both the WT and the *GmCBP60b.1*-overexpressing plants with the bacterial pathogen *Pseudomonas syrangae* pv. tomato DC3000 (*Pst* DC3000). As shown in Figure 4F, the multiplication of *Pst* DC3000 on the leaves of the *GmCBP60b.1*-overexpressing plants was drastically reduced compared with that on the WT plants, demonstrating that *GmCBP60b.1* plays a positive role in defense responses.

### 2.4. Transient Overexpression of GmCBP60b.1 Activates the Expression of a Luciferase Gene Driven by GmSARD1 Promoter, Which Contains Multiple CBP60b-Specifc Cis-Elements

**Figure 5:**
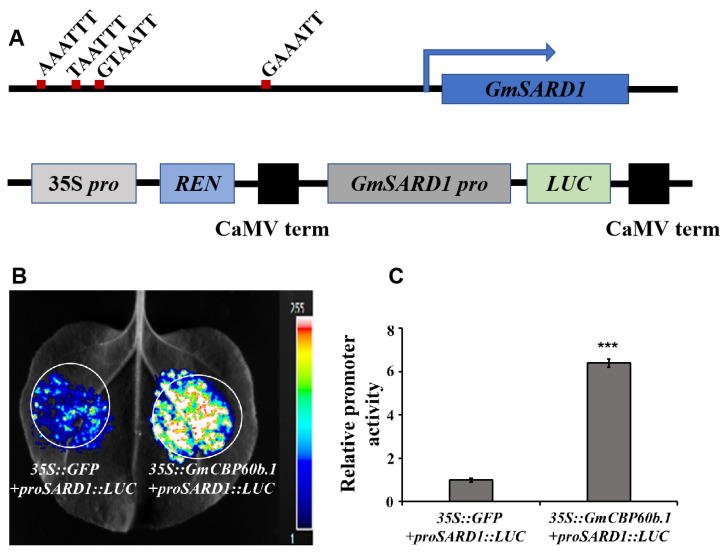
*Gm*CBP60b.1 activates the expression of LUC reporter gene driven by a 1240 bp promoter region of *GmSARD1*. (**A**) Identification of *Gm*CBP60b binding site in *GmSARD1* promoter region. Four motifs that differ by one nucleotide from G(A/T)AATT(T/G) were found in a 1240 bp promoter region of *GmSARD1*. The red box in the upper panel indicates the motif site. The schematic diagram of *proGmSARD1-LUC* fusion vector constructed by cloning the 1240-bp *GmSARD1* promoter fragment containing four motifs into *pGreenII0800-LUC* vector (lower panel). (**B**) *Gm*CBP60b.1 activates the expression of *proGmSARD1::LUC*. *proGmSARD1::LUC* and *35S::GmCBP60b.1* or *35S::GFP* and *35S::REN* expressing the Renilla luciferase (OD_600_ = 0.05) were transiently coexpressed in the leaves of *N. benthamiana* plants mediated by Agrobacterium infiltration. LUC luminescence was detected and captured using a microscope at 40 h post-infiltration (dpi). (**C**) LUC activity was measured quantitatively using the dual-luciferase reporter assay system. The ratio of firefly LUC/Renilla LUC represents relative promoter activity. Student’s *t*-test (*** *p* < 0.001).

In Arabidopsis, CBP60b binds to the core cis-element G(A/T)AATT(T/G) in the promoter regions of defense-related genes, such as *SARD1* and *ICS1*, and it activates their expression [11,12]. We found that there were four core cis-element-like motifs, (G/A/T)AATT, within a 1240 bp promoter region of *GmSARD1* (Figure 5A, upper panel). We reasoned that *Gm*CBP60b might be able to bind these motifs and thus activate the expression of *GmSARD1*. To test this possibility, we first cloned a 1240 bp fragment of the *GmSARD1*promoter into a pGreenII0800-LUC vector to generate a *proGmSARD1::LUC* construct (Figure 5A, lower panel). Next, we transiently coexpressed *35S::GmCBP60b.1* with *proGmSARD1::LUC* in the leaves of *N. benthamiana* plants via agro-infiltration. If our postulation is correct, the expression of the luciferase gene will be activated in the presence of *Gm*CBP60b in the infiltrated region. As expected, the expression of the luciferase gene was highly activated in the co-infiltrated area (Figure 5B, right), indicating that *Gm*CBP60b could bind to the (G/A/T)AATT motifs and activate the expression of the *LUC* reporter gene. Because the pGreenII0800-LUC vector also carries a *35S::Rluc* cassette, the relative promoter activity of the *proGmSARD1* could be normalized by Renilla luciferase, which serves as a control. As shown in Figure 5C, the relative promoter activity of *proGmSARD1* was significantly higher in the presence of *Gm*CBP60b.1 than in the presence of GFP, further confirming that *Gm*CBP60b.1 can activate the expression of the *LUC* reporter gene driven by the *GmSARD1* promoter. We found that the *LUC* reporter gene was also induced to a certain level in the area co-infiltrated with *proGmSARD1::LUC* and *35S::GFP* (Figure 5B, left), which could be activated by one or more endogenous CBP60b-like protein(s).

### 2.5. GmCBP60b.1-GFP Localizes in the Nucleus

**Figure 6:**
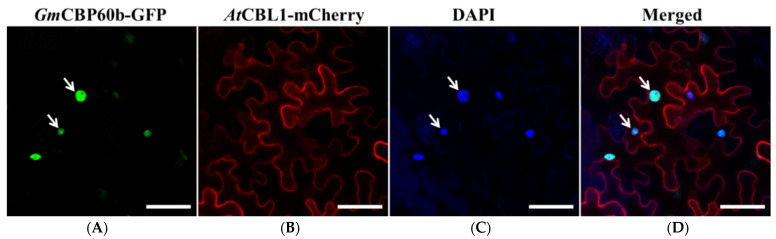
*Gm*CBP60b.1 is localized in the nucleus. (**A**) The green fluorescence emitted by GmCBP60b.1-GFP is localized in the nuclei (see arrows). (**B**) The red fluorescence emitted by *At*CBL1-mCherry is localized on the plasma membrane (PM). (**C**) The blue fluorescence of DAPI staining is localized in the nuclei. (**D**) Superposition of the green, red, and blue channels. Arrows point to the nuclei. Scale bar = 20 μm.

CBP60b is a transcription factor that is supposed to be localized in the nucleus. To experimentally prove this, *Gm*CBP60b.1 was N-terminally fused with GFP, and the resulting *35S::GmCBP60b.1-GFP* fusion construct was transiently coexpressed with *AtCBL1-mCherry* in the leaves of *N. benthamiana* plants via agro-infiltration, followed by observation under a confocal microscope. Consistent with its role as a transcription factor, *Gm*CBP60b.1-GFP signals were exclusively localized in the nuclei, which were colocalized with the blue signals specifically stained by DAPI (Figure 6, see arrows). As a control, *At*CBL1-mCherry was exclusively localized on the plasma membrane (PM).

## 3. Discussion

The CALMODULIN-BINDING PROTEIN 60 (CBP60) family in Arabidopsis is a small eight-member family of atypical transcription factors. Three members of the CBP60 family have been previously characterized. Two of them function as positive regulators (SARD1 and CBP60g) and the third one functions as a negative regulator (CBP60a) in defense responses [10]. Interestingly, two groups recently reported that the fourth member, CBP60b, functions as both a positive and negative regulator of Arabidopsis immunity [11,12].

CBP60g and SARD1 participate in Arabidopsis immunity by regulating the expression of genes in SA and NHP biosynthesis, as well as in the ETI, PTI, and SAR pathways, by directly binding to the core cis elements on their promoters [2,3,4,5,6,7].

It was unexpectedly found that CBP60b can activate the expression of *SARD1* and *CBP60g*, indicating that CBP60b is a transcription activator. Either the loss of function or overexpression of CBP60b results in similar autoimmunity, including dwarfism, the over-accumulation of ROS and SA, a highly induced expression of *PR* genes, and an enhanced resistance to the bacterium *Pst* DC3000 [11,12]. CBP60b probably participates in Arabidopsis immunity by directly regulating the expression of *CBP60g* and *SARD1* [11,12], both of which are required for the biosynthesis of SA/NPH, as well as the activation of defense-related genes [2,3,4,5,6,7]. Whereas the autoimmunity of *cbp60b* mutant could be fully rescued by EDS1 or PAD4 loss-of-function mutations, the autoimmunity resulting from *CBP60b* overexpression could not be rescued by the loss function of EDS1 or PAD4, suggesting that the autoimmunity displayed in knock-out and in overexpressing plants results from two different signaling pathways [11].

One may wonder how the loss of function and overexpression of a transcription activator can lead to the same autoimmune phenotype. The authors proposed that CBP60b may serve as a guardee based on the guard/decoy hypothesis [18,19,20]. The level of CBP60b proteins is monitored by the nucleotide-binding leucine-rich repeat receptor (NLR) surveillance system, and the absence of CBP60b can be detected by NLRs, which leads to the activation of ETI [11]. Huang et al. [12] proposed that, in addition to activating the expression of *SARD1*, CBP60b could also be required for the expression of an unknown gene that encodes a guardee/decoy or a negative regulator of a TNL(s). In the absence of CBP60b, either the absence of the protein encoded by the unknown gene is detected by NLR(s) or the inhibition of NLRs is released, which, in turn, activates ETI.

Consistent with the results in Arabidopsis, we found that either silencing *GmCBP60b1/2* in soybean or overexpressing *GmCBP60b1* in tobacco resulted in a similar autoimmunity (Figure 1, Figure 2, Figure 3 and Figure 4), indicating that the function of CBP60b homologues is highly conserved in different plant species. CBP60 transcription factors contain a highly conserved DNA-binding domain (DBD). *At*CBP60b1 exclusively localizes in the nuclei and activates the expression of defense-related genes, such as *ICS1*, *SARD1*, and *PRs*, by binding to a conserved core cis-element, G(A/T)AATT(T/G) [11,12]. Consistent with its role as a TF, *Gm*CBP60b was found to be exclusively localized in the nuclei (Figure 6). Even though the (A/T)AATT(T/G) core motif was not present within the 1240 bp *GmSARD1* promoter fragment (Figure 5A), *GmSARD1Pro::LUC* could still be activated by *Gm*CBP60b (Figure 5B,C). Accordingly, four similar motifs, (A/T)AATTT or G(A/T)AATT, with only one nucleotide difference with the core cis-element, G(A/T)AATT(T/G), were found in the 1240 bp *GmSARD1* promoter fragment (Figure 5A), suggesting that these motifs can still be recognized and bound by *Gm*CBP60b to activate the expression of *GmSARD1Pro::LUC* (Figure 5B,C). These results raise the possibility that a slight difference may exist between *At*CBP60b and *Gm*CBP60b in recognizing and binding to the core cis-elements.

Based on our own results presented in this report and the results reported in Arabidopsis, we proposed a model to explain the positive and negative regulatory roles of *Gm*CBP60b in immunity. In wild-type soybean plants, the expression of *GmCBP60b* is low, and the expression of immune-related genes is not induced. As a result, no defense responses are activated. In *GmCBP60b*-overexpressing tobacco plants, the over-accumulated *Gm*CBP60b binds to the cis-elements of immune-related genes (SA/NHP biosynthesis genes, *CBP60g/SARD1*, and SAR-related genes) and activates the expression of these genes. As a result, the biosynthesis of SA and NHP is significantly enhanced, and immune responses are activated, which leads to a dwarfed stature and spontaneous cell death on the leaves. In *GmCBP60b*-silenced soybean plants, the significantly reduced *Gm*CBP60b level is either detected by an unidentified NLR(s) [11] or results in a reduced expression of a guardee/decoy or a negative regulator of immunity mediated by TIR (N-terminal Toll-interleukin-1 receptor-like)-domain NLRs [12]. Because the execution of the activated immunity depends on the intact defense signaling pathway, the loss function of any of the downstream proteins, such as EDS1 and PAD4, can abolish the autoimmunity caused by *GmCBP60b* silencing [11,12].

Increasing evidence suggests that either the loss of function or overexpression of a defense-related gene, such as MEKK1 [21,22], MPK6 [23], Senescence-Associated E3 Ubiquitin Ligase 1 (SAUL1) [24,25,26], and CBP60b [11,12], leads to autoimmunity. These proteins are guarded by NLR proteins [24,27]. Either the loss/disappearance or over-accumulation of these guardees can lead to the activation of NLR-mediated ETI, which depends on EDS1 and PAD4 [24,27].

## 4. Materials and Methods

### 4.1. Plant Materials

Soybean (*Glycine max*, cv. Williams 82) and *Nicotiana tabacum* cv. Samsun were used in this study. Soybean and tobacco seeds were firstly germinated in soil in a small pot. After 15 days, the soybean and tobacco seedlings were transferred to bigger pots. The soybean and tobacco plants were kept in a growth chamber or growth room at 22 °C (photoperiod: 16 h light/8 h dark).

### 4.2. BPMV-Mediated VIGS

The BPMV-VIGS system has been described previously [15,16]. The orthologs of *GmCBP60b* were identified by a BLASTn search of the soybean genome in the Phytozome database (www.phytozome.org, last accessed on 1 June 2019) using the Arabidopsis (*Arabidopsis thaliana*) *CBP60b* (AT5G57580) sequence. A 303 bp fragment of *GmCBP60b1* (*Glyma.*17G065500) was amplified via PCR using the following primers: *GmCBP60b1*-F: 5′-aag**GGATCC**GTGGTAAAAGAACGTGAAGGA-3′, and *GmCBP60b1*-R: 5′-ttg**GGTACCC**ACTGGGTAGGAACGGATAG-3′. The bold sequences are *Bam*HI and *Kpn*I restriction sites, respectively. The PCR fragment amplified using the above-mentioned primer pair was cloned into the BPMV-VIGS (IA-D35) vector via double digestion with the same pair of restriction enzymes. The boldface letter indicates the extra nucleotide in reverse primers needed to maintain the reading frame. The following primers were used for silencing verification and internal control:

*GmCBP60b.1/2-V*-F: aaaTATTCACAGGAGGAAAAGTGG;

*GmCBP60b.1/2-V*-R: tttGAGAGAATCAGCAGAATAGAATTG;

*NtUBQ5*-F: CCTAACGGGTAAAACAATCAC;

*NtUBQ5*-R: AGCCATAAAAGTTCCAGCAC.

### 4.3. RNA Extraction and RT-qPCR

Total RNA was extracted with Trizol (Invitrogen, Waltham, MA, USA). RT-qPCR was performed as described [22] using the 2× SYBR Green qPCR Mix (Aidlab, Beijing, China) and an ABI550 Real-Time PCR machine (Applied Biosystems, Thermo Fisher Scientific, Austin, TX, USA). The following primers were used:

qRT-*NtUBQ5*-F: TCTTCATCTCGTGCTCCG;

qRT-*NtUBQ5*-R: TCCAGAATCATCCACCTTGTA;

qRT-*NtPR1*-F: GCTGAGGGAAGTGGCGATTTC;

qRT-*NtPR1*-R: CCTAGCACATCCAACACGAACC;

qRT-*NtPR3*-F: GACCCATCCAATTGACAAACCAA;

qRT-*NtPR3*-R: CTGTGGTGTCATCCAGAACC;

qRT-*NtPR5*-F: GGCGATTGTGGCTCAAACC;

qRT-*NtPR5*-R: GAAATCTTGCTTCGTACCTGAGA.

### 4.4. Pseudomonas syringae pv. glycinea (Psg) R4 and P. syringae pv. Tomato DC3000 Growth Assays for the Infected Soybean and Tobacco Leaves

*Pst* DC3000 (OD_600_ = 0.00001) was injected into the leaves of different tobacco lines as described [28]. Leaf discs (0.5 cm in diameter) were collected from the infiltrated leaf areas and ground in an extraction buffer (10 mM MgSO_4_). The extracted saps were diluted by the factors of 10, 100, and 1000 and spread on plates with KB agar medium. Colony-forming units (cfu) was calculated by the number of colonies on a series of plates with different dilution factors.

### 4.5. SMV-N-GUS Inoculation, GUS Staining, and GUS Foci Measurements

SMV-N-GUS inoculation, GUS staining, and GUS foci measurements were described previously [17]. After SMV-N-GUS infection, the leaves were detached and incubated for 3 days on a piece of moist filter paper in a Petri dish. GUS staining was then performed as described [29]. The leaves were photographed using a stereomicroscope. The diameters of GUS foci were measured using Image-Pro Plus 6.0 (Media Cybernetics, Rockville, MD, USA).

### 4.6. H_2_O_2_ Detection by 3,3′-Diaminobenzidine Staining

H_2_O_2_ was detected using the DAB staining method as described previously [30].

### 4.7. SA Quantification

SA was quantified using an Agilent 1260 HPLC system (Agilent Technologies, Santa Clara, CA, USA) with a diode array detector, a fluorescence detector, and a column, as described previously [31].

### 4.8. Generation of Transgenic N. tabacum Samsun (NN) Plants That Overexpress GmCBP60b1

The full-length cDNA of *GmCBP60b1* was amplified using the following pair of primers with KpnI and XbaI sites (the sequences in bold) attached to the 5′ end and 3′ end, respectively:

*GmCBP60b1-pE1776*-F: aaa**GGTACC**ATGAAGAAACCAACAA;

*GmCBP60b1-pE1776*-R: ttt**TCTAGA**TTATTCATCTAACTCCTCTATCTG.

The amplified fragment was double-digested with KpnI and XbaI and subsequently cloned into the pE1776 vector [32] predigested with the same pair of restriction enzymes, followed by ligation and transformation. *pE1776-GmCBP60b1* was transformed into the *Agrobacterium* strain GV3101 and then transformed into *N. tabacum* Samsun (NN) following a protocol described previously [33].

### 4.9. Subcellular Localization of GmCBP60b1

The open reading frame (ORF) of *GmCBP60b1* was amplified from cDNA transcribed from total RNA extracted from tobacco plants using the *GmCBP60b.1*-*KpnI*-F: (aaa**GGTACC**ATGAAGAAACCAACAACCGA) and *GFP-GmCBP60b.1*-R: (GGAGCCACCGCCACCAGAGCCACCACCGCCTTCATCTAACTCCTCTATCTGTGC) primer pair, and GFP was amplified using the *GmCBP60b.1*-*GFP*-F (GGCGGTGGTGGCTCTGGTGGCGGTGGCTCCATGGTGAGCAAGGGCGA) and *GFP*-XbaI-R (ttt**TCTAGA**TTACTTGTACAGCTCGTCCATGC) primer pair. *GFP-GmCBP60b.1*-R and *GmCBP60b.1*-*GFP*-F primers are complementary to each other. The *GmCBP60b.1*-*GFP* fusion was subsequently amplified by mixing the *GmCBP60b1* and *EGFP* PCR products amplified above by overlapping the PCR approach using the *GmCBP60b.1*-*KpnI*-F and *EGFP*-*XbaI-R* primer pair. The amplified fusion PCR product was purified and double-digested with KpnI and XbaI and then cloned into the pE1776 binary vector [33]. The bold letters in the primers represent the KpnI and SacI restriction sites.

### 4.10. Agro-Infiltration and Confocal imaging

This fusion construct was transformed into the *Agrobacterium* GV3101 strain and subsequently infiltrated into tobacco leaves as described [22]. The infiltrated leaf area was excised at 2 days post-infiltration and was observed under a confocal laser-scanning microscope (LSM 880, Zeiss, Jena, Germany).

### 4.11. Luciferase Dual-Report Assay

A dual-reporter assay was performed with the reporter constructs as described [11]. A 1240 bp promoter fragment of *GmSARD1* was amplified from soybean genomic DNA using the following pair of primers (the bold sequences represent HindIII and BamHI restriction sites, respectively):

*ProGmSARD1*-HindIII-F: ccc**AAGCTT**GACAACGTTACATTGAATTAACAG;

*ProGmSARD1*-BamHI-R: cgc**GGATCC**TGGTGAAGAATCTGGTAA.

The amplified fragment was purified and digested with HindIII and BamHI and subsequently cloned into the pGreen Ⅱ0800-Luc vector [34] pre-digested with the same set of enzymes. A 35S promoter-driven Renilla luciferase reporter carried on the pGreen Ⅱ0800-Luc vector served as an internal control. *Agrobacterium* carrying pGreen Ⅱ0800-Luc-p*GmSARD1*::Luc and 35S::Renilla was infiltrated into the leaves of *N*. *benthamiana* plants with a final concentration of OD_600_ = 0.2. To visualize and image the luminescence intensity, *N*. *benthamiana* leaves were infiltrated with 100 mM D-luciferin at 2 days post-inoculation with the Agrobacterium and then imaged under a low-light cooled CCD imaging apparatus (Tanon 6600, Biotanon, Shanghai, China). LUC activity was measured with a Dual-LUC Reporter Assay Kit (Promega, Beijing, China). Relative promoter activities were calculated as the ratio of firefly luciferase/Renilla luciferase [11].

## Data Availability

Data are contained within the article.

## Abbreviations

CBP60, CALMODULIN-BINDING PROTEIN 60; SARD1, SAR DEFICIENT 1; VIGS, virus-induced gene silencing; PAMPs, pathogen-associated molecular patterns; PTI, PAMP-triggered immunity; ETI, effector-triggered immunity; SA, salicylic acid; NHP, *N*-hydroxy-pipecolic acid.
